# Severe hepatotoxicity associated with orlistat: clinical presentation, risk factors and outcomes

**DOI:** 10.3389/fphar.2026.1811846

**Published:** 2026-07-02

**Authors:** Zhuangzhuang Ma, Zhensen Niu, Aiyan Yu, Yuxin Guo, Tianping Chen, Naiju Zhang

**Affiliations:** 1 Department of Pharmacy, The First Affiliated Hospital of Bengbu Medical University, Anhui Engineering Technology Research Center of Biochemical Pharmaceutical, Institute of Emergency and Critical Care Medicine, Branch of National Clinical Research Center for Infectious Diseases, Bengbu, China; 2 Department of Rehabilitation Medicine, Bengbu Infectious Disease Hospital, Bengbu, China; 3 Department of Cardiology, The First Affiliated Hospital of Bengbu Medical University, Bengbu, Anhui, China

**Keywords:** acute liver failure, alcohol, drug-induced liver injury, elderly, orlistat, risk stratification

## Abstract

**Background and Aims:**

Orlistat, a widely prescribed anti-obesity agent, has been associated with rare but severe hepatotoxicity. The risk profile remains poorly characterised in vulnerable populations, particularly elderly individuals and those with concurrent alcohol use. This study presents a fatal case of OILI and systematically reviews published cases to delineate the clinical spectrum of the injury and highlight features that may be relevant to vulnerable populations.

**Methods:**

We report a fatal case of OILI in a 66-year-old man with chronic alcohol use. A systematic literature review was conducted across PubMed, Web of Science, Embase and CNKI up to December 2025 to identify all published cases of OILI. Data on demographics, drug exposure, clinical presentation, laboratory values, and outcomes were extracted and analysed descriptively.

**Results:**

Ten cases were analyzed. The median age was 55.5 years (range: 15–66), with a female predominance (80%). Hepatocellular injury was the dominant pattern (nine out of ten). Severe outcomes, including acute liver failure, occurred in 60% (six out of ten) of patients, with a mortality rate of 20% (two out of ten) and 30% (three out of ten) requiring liver transplantation. Both fatalities were males aged ≥60 with alcohol use. Risk factors for severe outcomes included age ≥50 years, alcohol use, and duration of orlistat use >8 weeks. In patients with poor outcomes, transaminases often exceeded 5,000 U/L, bilirubin rose above 171 μmol/L, and INR was greater than 2.0.

**Conclusion:**

OILI can present with acute liver failure, particularly in elderly patients with alcohol use. These observations suggest that close monitoring and prompt intervention may be important in high-risk patients, though larger studies are needed to confirm.

## Highlights

- Orlistat causes acute liver failure in elderly patients with alcohol use.
- Age ≥50, alcohol, >8 weeks use predict severe hepatotoxicity.
- Risk-stratified monitoring and early transplant referral are essential.

## 1 Introduction

Orlistat, a gastrointestinal lipase inhibitor, is a currently available non-prescription weight-loss medication for long-term use, which, when combined with lifestyle interventions, produces modest but definite weight-loss effects (Kaur et al., 2025). Although generally considered safe, orlistat has been associated with rare but severe hepatotoxicity in post-marketing surveillance, prompting regulatory warnings from agencies like the U.S. Food and Drug Administration (FDA). The clinical presentation of Orlistat-Induced Liver Injury (OILI) is variable, ranging from asymptomatic transaminase elevation to fulminant hepatic failure (Sall et al., 2014).

The pathophysiological mechanisms remain incompletely understood, with proposed hypotheses including idiosyncratic metabolic reactions, though direct evidence is lacking. Identifying susceptible individuals is a significant clinical challenge. Epidemiological data and case reports suggest that advanced age and concomitant alcohol use may synergistically increase risk, likely through age-related declines in hepatic regenerative capacity, drug metabolism, and glutathione reserves, compounded by alcohol-induced oxidative stress and mitochondrial dysfunction.

Despite these concerns, the clinical profile, risk factors, and optimal management strategy for OILI, especially in high-risk populations, have not been systematically synthesized from available case evidence. This study aims to fill this gap by presenting a detailed fatal case of OILI in an elderly patient with alcohol use and integrating it with a systematic review of published literature. Our objectives are to: (1) describe the clinical and biochemical spectrum of OILI; (2) explore potential demographic and clinical risk factors for severe outcomes; and (3) propose a structured, evidence-informed framework for prevention, monitoring, and management.

## 2 Materials and methods

### 2.1 Case report

We report a case of fatal acute liver failure following orlistat use, managed at The First Affiliated Hospital of Bengbu Medical University. The collection and publication of anonymized clinical data were approved by the hospital’s Institutional Ethics Committee (protocol code 2020KY072 and date of approval: 3 August 2020).

### 2.2 Systematic literature review

**FIGURE 1:**
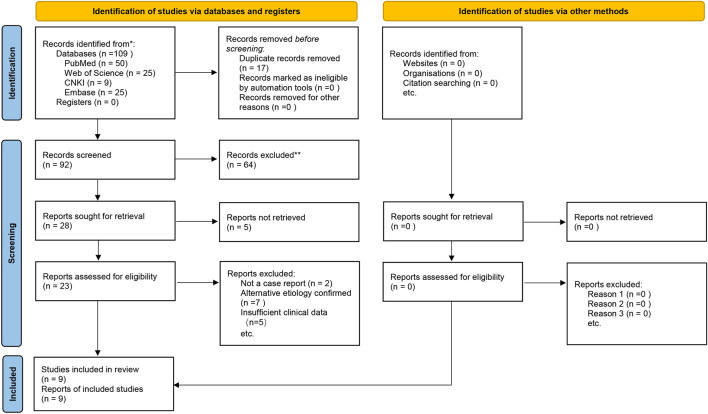
PRISMA 2020 flow diagram. Records were identified from PubMed (n = 50), Web of Science (n = 25), CNKI (n = 9), and Embase (n = 25). After deduplication (n = 17), 92 records were screened by title and abstract, with 50 excluded (37 not case reports, 13 confirmed alternative etiology). Full texts of the remaining 42 records were sought; five were unobtainable, leaving 37 for eligibility assessment. After full-text review, 28 were excluded, and 9 case reports were included. No records were identified from other sources.

A systematic literature review was conducted in accordance with the Preferred Reporting Items for Systematic Reviews and Meta-Analyses (PRISMA) guidelines (see Figure 1). To identify all relevant case reports, we comprehensively searched four electronic databases: PubMed, Web of Science, Embase, and CNKI. The search encompassed all records from database inception until 20 March 2026. The search strategy was designed to combine terms related to orlistat with terms related to liver injury. In PubMed, a combination of Medical Subject Headings (MeSH) terms and free-text keywords was used. The core search strategy for PubMed is presented below as an example: [(“Orlistat” [Mesh]) OR (Orlistat) OR (Xenical)] AND [(“Chemical and Drug Induced Liver Injury” [Mesh]) OR (liver injury) OR (hepatotoxicity) OR (hepatitis) OR (acute liver failure)] Similar search concepts were adapted using appropriate syntax and controlled vocabularies for Web of Science, Embase, and CNKI. For Embase, the search was optimized using Emtree terms and adverse drug reaction-specific subheadings to maximize sensitivity for pharmacovigilance data. No filters were applied for language or publication type during the initial search to maximize sensitivity. Additionally, the reference lists of all retrieved full-text articles were manually screened to identify any potentially eligible reports not captured by the electronic search. Inclusion criteria were: (1) were case reports or case series describing liver injury in patients taking orlistat; (2) provided sufficient clinical, laboratory, and temporal data to allow for an assessment of causality and clinical outcome; (3) were published in English or Chinese. Exclusion criteria were: (1) were conference abstracts, reviews, or commentaries with insufficient original patient data; (2) described cases where an alternative etiology (e.g., viral hepatitis, autoimmune hepatitis, other hepatotoxic drugs); was conclusively established as the predominant cause of liver injury. The study selection process was performed independently by two reviewers. First, titles and abstracts were screened against the eligibility criteria. Subsequently, the full texts of potentially relevant articles were assessed. Any discrepancies between reviewers were resolved through discussion or, if necessary, consultation with a third reviewer.

### 2.3 Data extraction and analysis

Data were extracted into a standardized form, including: demographics, medical history, orlistat dosage and duration, clinical presentation, peak laboratory values (alanine aminotransferase (ALT), aspartate aminotransferase (AST), Total bilirubin (TBil), international normalized ratio (INR), causality assessment score (e.g., the Roussel Uclaf Causality Assessment Method (RUCAM) (see Table 1), liver histology, key interventions, and clinical outcome. Given the nature of the data (case reports/series), the analysis was primarily descriptive, focusing on frequencies, proportions, medians, and ranges. Trends in potential risk factors for severe outcomes (liver failure, death, transplantation) were identified through comparative case analysis.

**TABLE 1:** RUCAM score assessment for the present case.

Scoring item	Score	Explanation
1. Time to onset	+2	Onset occurred within 90 days of drug initiation, clearly consistent with drug-induced liver injury.
2. Course (After withdrawal)	+1	The ALT decline >50% within 8 days was confounded by concurrent plasma exchange, limiting attribution to drug withdrawal alone; the ALT decline >50% within 8 days cannot be solely attributed to drug withdrawal.
3. Risk factors	+1	Long-term alcohol use history (40 years) was clearly present, increasing susceptibility.
4. Exclusion of other causes	+2	Viral hepatitis (HAV, HBV, HCV, HEV), autoimmune hepatitis, and other potential etiologies were comprehensively excluded.
5. Known hepatotoxicity	+1	Hepatotoxicity risk is clearly documented in the drug label and previous case reports.
6. Rechallenge	0	Rechallenge was not performed.
Total score	7	Causality level: Probable

### 2.4 Quality assessment

The methodological quality of the included case reports was independently assessed by two reviewers using the Joanna Briggs Institute (JBI) Critical Appraisal Checklist for Case Reports (see Table 2). This standardized tool consists of eight items that evaluate key methodological domains: (1) Were the patient’s demographic characteristics clearly described? (2) Was the patient’s history clearly described and presented as a timeline? (3) Was the current clinical condition of the patient on presentation clearly described? (4) Were diagnostic tests or assessment methods and the results clearly described? (5) Was the intervention(s) or treatment procedure(s) clearly described? (6) Was the post-intervention clinical condition clearly described? (7) Were adverse events (harms) or unanticipated events identified and described? (8) Does the case report provide takeaway lessons? Each item was rated as “yes” (clearly reported and adequate), “no” (not reported or inadequate), or “unclear” (insufficient information to make a judgment). A total score was calculated by summing the number of “yes” responses, with a maximum possible score of 8. Higher scores indicate better methodological quality.

**TABLE 2:** Methodological quality of included case reports (JBI checklist).

No.	First author (Year)	Q1	Q2	Q3	Q4	Q5	Q6	Q7	Q8	Total
1	Present case (2026)	Y	Y	Y	Y	Y	Y	Y	Y	8
2	Li et al. (2022)	Y	Y	Y	Y	Y	Y	Y	Y	8
3	Qiu et al. (2019)	Y	Y	Y	Y	Y	Y	Y	Y	8
4	Kim et al. (2002)	Y	Y	Y	Y	Y	Y	Y	Y	8
5	Montero et al. (2001)	Y	Y	Y	Y	Y	Y	U	Y	7
6	Thurairajah et al. (2005)	Y	Y	Y	Y	Y	Y	Y	Y	8
7	Sall et al. (2014)	Y	Y	Y	Y	Y	Y	Y	Y	8
8	Umemura et al. (2006)	Y	Y	Y	Y	Y	Y	U	Y	7
9	Lau and chan (2002)	Y	Y	Y	Y	Y	Y	Y	Y	8
10	Wilson et al. (2011)	Y	Y	Y	U	Y	Y	U	Y	6

(1) Were the patient’s demographic characteristics clearly described?

(2) Was the patient’s history clearly described and presented as a timeline?

(3) Was the current clinical condition of the patient on presentation clearly described?

(4) Were diagnostic tests or assessment methods and the results clearly described?

(5) Was the intervention(s) or treatment procedure(s) clearly described?

(6) Was the post-intervention clinical condition clearly described?

(7) Were adverse events (harms) or unanticipated events identified and described?

(8) Does the case report provide takeaway lessons?

“Y” = yes (1 point), “N” = no (0 points), and “U” = unclear (0 points). Q1–Q8 correspond to the eight items of the JBI, critical appraisal checklist for case reports.

## 3 Results

### 3.1 Case presentation

On 4 January 2021, a 66-year-old man was admitted to the hospital after taking oral orlistat at a dosage of 120 mg three times daily for 3 months to treat his obesity. No routine liver function tests were performed during this period. He had a 40-year history of consuming 150 mL of 56-proof white wine daily, with his most recent drink occurring just 1 day before his hospital admission.

Three days before admission, he experienced dizziness, fatigue, dark urine, and fever (Tmax 38 °C). Within 48 h of admission, mild icterus of the skin and sclerae was noted, with palmar erythema and spider angiomas absent. The patient was positive for hepatic percussion tenderness, the liver was not palpable for enlargement, and shifting dullness was negative. Subsequently, mild confusion and lethargy developed, a Glasgow Coma Scale (GCS) score of 3, asterixis could not be assessed due to the comatose state. Between January 4 and 12, ALT levels fell sharply from 8410 U/L to 39 U/L, while TBil concurrently rose to 319.7 μmol/L, accompanied by progressively declining albumin and a significantly elevated INR.

Orlistat and alcohol were identified as potential contributors to the patient’s liver injury, prompting their immediate withdrawal. Subsequently, hepatoprotective therapy was initiated, incorporating glycyrrhizin and Transmetil. Despite these interventions, the patient’s condition did not improve. By January 6, he exhibited grade IV hepatic encephalopathy and declining coagulation status, leading to the initiation of plasma exchange therapy.

**FIGURE 2:**
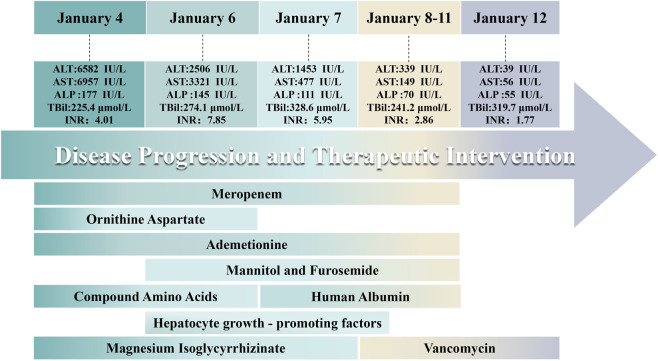
Clinical course and therapeutic interventions in the patient. ALT, alanine aminotransferase; AST, aspartate aminotransferase; ALP, alkaline phosphatase; TBil, total bilirubin; INR, international normalized ratio.

Throughout the course of plasma exchange, the patient developed multiple organ dysfunction syndrome (MODS), characterized by acute renal failure and septic shock. To address these critical issues, a comprehensive treatment plan was implemented, including mechanical ventilation, administration of broad-spectrum antibiotics to combat septic shock, the use of vasopressors for hemodynamic support, as well as mannitol, lactulose, and continuous renal replacement therapy (CRRT) for renal failure. Despite these aggressive interventions, the patient’s condition continued to deteriorate irreversibly, resulting in his passing on hospital day 8, January 12 (Figure 2).

### 3.2 Literature case series analysis

A total of nine published cases met the inclusion criteria, which, when combined with our own case, resulted in a cohort of ten patients (see Table 3) (Sall et al., 2014; Li et al., 2022; Qiu et al., 2019; Kim et al., 2002; Montero et al., 2001; Thurairajah et al., 2005; Umemura et al., 2006; Lau and Chan, 2002; Wilson et al., 2011). The median age of the cohort was 55.5 years, with a range from 15 to 66 years, and a predominant representation of females, accounting for 80% (eight out of ten patients). The median latency period until symptom onset was 8.5 weeks, with a range spanning from 4 days to 13 months. The injury pattern observed in these patients was predominantly hepatocellular, affecting nine out of ten cases.

**TABLE 3:** Clinical characteristics, management, and outcomes of patients with orlistat-associated liver injury.

No.	References (Year)	Age/ Sex	Medical history	Dosage/ Duration	Onset	RUCAM score	Clinical manifestations	Laboratory index (peak)	Pathology	Key treatment	Outcome
1	Present case (2026)	66/ M	Obesity, chronic alcohol use	120 mg TID, 3 months	3 months	7	Dizziness, fatigue, dark urine, fever, jaundice, hepatic encephalopathy, coma	ALT: 8410 U/L, AST: 8500 U/L, TBil: 319.7 μmol/L, INR: 7.85, scr: 193 μmol/L	Not provided	Orlistat discontinued; hepatoprotective agents (glycyrrhizin, ademetionine); plasma exchange; CRRT; mechanical ventilation	Death
2	Li et al. (2022)	37/ F	None	120 mg TID, 4 weeks	4 weeks	5	Abdominal pain, steatorrhea, fatigue, anorexia, nausea, jaundice, oliguria	ALT: 12,371 U/L, AST: 15,830 U/L, TBil: 54.5 μmol/L, scr: 215 μmol/L	Ultrasound: coarse liver echo	Orlistat discontinued; compound glycyrrhizin, glutathione, polyene phosphatidylcholine	Recovery
3	Qiu et al. (2019)	58/ F	Hypertension, CAD	0.12 g OD, 13 months	13 months	8	Fatigue, poor appetite	ALT: 1,603 U/L, AST: 1,265 U/L, TBil: 21.8 μmol/L	Not provided	Orlistat discontinued; diammonium glycyrrhizinate, then magnesium isoglycyrrhizinate	Recovery
4	Kim et al. (2002)	33/ F	None	120 mg TID, 2 months	2 months	CDS: 14	Jaundice, malaise, fatigue, anorexia, nausea, dyspepsia	ALT: 251 IU/L, AST: 98 IU/L, TBil: 20.1 mg/dL	Liver biopsy: canalicular cholestasis, hepatocyte ballooning, spotty necrosis	Orlistat discontinued; steroid therapy	Improved
5	Montero et al. (2001)	35/ F	None	120 mg/day, 3 weeks	3 weeks	–	Ascites, hyponatremia, grade II encephalopathy	ALT: 1548 U/L, AST: 1132 U/L, TBil: 9.5 mg/dL, PT: 30%	Not provided	Orlistat discontinued; orthotopic liver transplantation	Recovered post-transplant
6	Thurairajah et al. (2005)	57/ F	Obesity (BMI 43)	120 mg TID, 12 weeks	12 weeks	–	Jaundice, malaise, nausea, ascites, encephalopathy	ALT: 1505 U/L, TBil: 138 μmol/L, INR: 1.7	Liver biopsy: bridging and lobular necrosis	Orlistat discontinued; liver transplantation	Submassive necrosis (post-transplant)
7	Sall et al. (2014)	54/ F	Hypertension, alcohol use	60 mg TID, 2 months	2 months	–	Fatigue, jaundice, pruritus, confusion, asterixis	ALT: 1942 U/L, AST: 2195 U/L, TBil: 19.2 mg/dL, INR: 3.0	Liver biopsy: necrotic hepatic parenchyma, drug toxicity likely	Orlistat discontinued; orthotopic liver transplantation	Recovered post-transplant
8	Umemura et al. (2006)	15/ F	None	120 mg TID, 7 days	7 days	–	Abdominal pain, malaise, nausea, diarrhea	ALT: 9976 IU/L, AST: 8269 IU/L, INR: 2.98	Not provided	Orlistat discontinued; intravenous vitamin K	Recovery
9	Lau and chan (2002)	62/ M	Hypertension, occasional alcohol	120 mg TID, 10 days	10 days	–	Hepatic failure	Progressive liver deterioration, coagulopathy	Autopsy: massive hepatocellular necrosis, bilirubinostasis	Supportive care	Death
10	Wilson et al. (2011)	40/ F	None	60 mg/day, 4 days	4 days	Naranjo: 3	Fulminant hepatic failure	Severe cholestasis, coagulopathy	Not provided	Orlistat discontinued; orthotopic liver transplantation	Liver transplantation

Severe clinical outcomes were common within this cohort, with acute liver failure developing in six patients (60%). Among these six, three went on to receive successful liver transplants, while two unfortunately succumbed to their condition, and one achieved recovery following transplantation. The overall mortality rate within this group was 20% (two out of ten), and the rate of liver transplantation was 30% (three out of ten).

In this series, the six cases with severe outcomes (liver failure, death, or transplant) shared some common features: all patients were ≥35 years old (four out of ten were ≥50), and prolonged orlistat use (>8 weeks) was commonly reported. Notably, both fatal cases were in males over 60 with a history of alcohol use. In contrast, the four patients who recovered fully without transplantation were younger (median age 37 years), and none had documented alcohol use. While these patterns cannot establish causality, they suggest that older age, prolonged use, and concomitant alcohol intake warrant further investigation as potential contributors to severe outcomes.

#### 3.2.1 Laboratory parameters and outcomes

**FIGURE 3:**
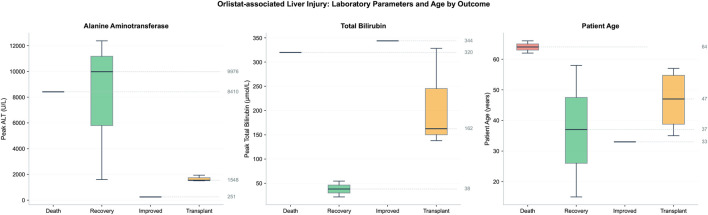
Distribution of peak laboratory parameters (Alanine Aminotransferase, Total Bilirubin) and Patient Age across cases. Values are presented on continuous scales (0.0–14000.0 for ALT and 0.0–350.0 for TBil; 0.0–100.0 for Age) to illustrate ranges and potential groupings.

Analysis of the laboratory profiles revealed heterogeneous injury patterns, with some patients showing profound transaminitis (peak ALT often exceeding 8,000 U/L) and others showing more pronounced cholestatic features (peak total bilirubin often exceeding 300 μmol/L). This heterogeneity suggests multiple underlying injury pathways rather than a single mechanism. In patients who died or required transplantation, transaminases often exceeded 5,000 U/L, total bilirubin rose above 171 μmol/L, and INR was greater than 2.0 (Figure 3). These values are presented as descriptive observations to illustrate the range of laboratory abnormalities in this small cohort; formal statistical comparisons between outcome groups were not performed due to the limited sample size, and these observations should not be interpreted as validated prognostic thresholds.

#### 3.2.2 Treatment patterns and clinical outcomes

**FIGURE 4:**
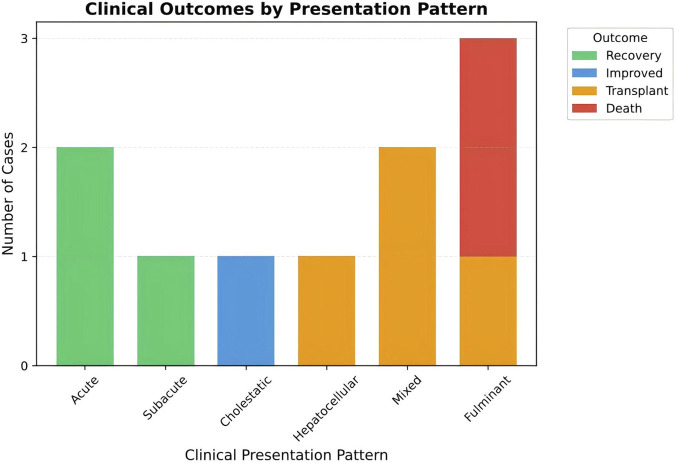
Clinical outcomes stratified by the presenting phenotypic pattern of orlistat-induced liver injury.

The analysis of management outcomes delineates a clear severity-dependent pathway. Patients with mild-to-moderate injury uniformly recovered after drug discontinuation and supportive care. In contrast, all severe cases met criteria for acute liver failure, with 40% (four out of ten) requiring orthotopic liver transplantation and a 20% (two out of ten) mortality rate, underscoring the life-threatening potential of severe OILI (Figure 4).

## 4 Discussion

This systematic review, combining a new fatal case with nine previously published reports, describes the clinical features of ten patients with severe OILI. Of the ten patients, the majority presented with hepatocellular injury, and 60% experienced severe outcomes (acute liver failure, death, or transplantation). Both fatalities were males aged over 60 with documented alcohol use.

These findings exist within a broader body of evidence on orlistat and liver safety. The largest epidemiological study to date, a self-controlled case series by Douglas et al. involving 94,695 orlistat users in United Kingdom primary care, found no evidence of increased acute liver injury risk during treatment. (Douglas et al., 2013). In that study, liver injury rates were elevated both before and after treatment initiation, suggesting that the observed association may be explained by underlying health conditions rather than the drug itself. This interpretation aligns with statements from the FDA and EMA, which have noted that a causal relationship has not been established.

Acknowledging the apparent discordance between these population-level findings and the severe cases documented in case reports is important. While population-level evidence suggests that orlistat does not increase the overall risk of acute liver injury, the cases summarized here illustrate that in a subset of patients, potentially those with underlying susceptibilities such as advanced age, chronic alcohol use, or other unidentified factors, severe idiosyncratic reactions may occur. This paradox highlights the limitation of epidemiological studies in detecting extremely rare events. The present series does not attempt to establish causality but instead describes the clinical features of severe cases and identifies patterns that merit further investigation.

Analysis of laboratory profiles revealed heterogeneous injury phenotypes, ranging from profound transaminitis to marked cholestasis, suggesting multiple underlying pathways. In patients with poor outcomes, transaminases often exceeded 5,000 U/L, bilirubin rose above 171 μmol/L, and INR was greater than 2.0. These values are consistent with severe hepatocellular injury as described in general drug-induced liver injury literature, such as Hy’s Law (Reuben, 2004) (ALT/AST >3× ULN concurrent with TBil >2× ULN) and dynamic models like the Model for End-Stage Liver Disease (MELD) score (Morales-Arráez et al., 2022), and illustrate the severity of biochemical derangement in the reported cases.

The analysis of our case series further highlights features that appeared repeatedly in patients with poor outcomes. Prolonged orlistat use (>8 weeks) was common, and both fatalities were males over 60 with documented alcohol consumption. These observations raise the possibility that aging and alcohol use may increase susceptibility to severe liver injury. However, given orlistat’s minimal systemic absorption (1%–3%), direct hepatotoxicity is pharmacologically difficult to reconcile. Alternative explanations such as unmasking of underlying liver conditions or patient-specific factors remain speculative and beyond the scope of this descriptive case series (Samiec et al., 1998; Crabb and Liangpunsakul, 2007; Cederbaum et al., 1974).

From a clinical management perspective, the index case illustrates a critical failure at the point of prescribing: despite the patient’s advanced age and heavy alcohol use, both known risk factors for liver injury, no baseline liver function tests were obtained, no risk stratification was performed, and no monitoring plan was established during 3 months of orlistat use. This absence of basic precautions allowed the liver injury to progress silently to irreversible failure. In contrast, patients with mild-to-moderate injury in the literature series uniformly recovered after drug discontinuation and supportive care. While no evidence-based monitoring protocol can be derived from a small case series, the experience underscores that pre-prescription risk assessment (including screening for alcohol use and baseline liver tests) and early recognition of suspected drug-induced liver injury are essential to prevent severe outcomes. N-acetylcysteine may be considered in acute liver failure, and timely evaluation for liver transplantation remains critical for patients who meet criteria.

Specifically, clear criteria for hospital admission should guide escalation. Hospital admission becomes mandatory if the patient shows signs of encephalopathy, an INR greater than 1.5, symptomatic jaundice, or a total bilirubin level above twice the upper limit of normal. The present patient delayed seeking care for 3 days and was admitted only after progressing to grade IV encephalopathy, demonstrating how late presentation drastically narrows the window for effective intervention. The fatal outcome described here was not an unavoidable idiosyncratic reaction but the consequence of sequential failures in risk assessment, monitoring, and timely intervention.

Our study has several limitations. The small sample size precludes definitive risk factor analysis or incidence estimation. Publication bias tends to favor the reporting of severe cases, and the presence of potential confounders, particularly alcohol use, makes it difficult to isolate the role of orlistat. Causality cannot be determined from case reports alone. Therefore, our findings should be interpreted as descriptive observations that generate hypotheses, not as definitive evidence of causation or validated risk factors.

In conclusion, this systematic review of ten cases describes the clinical features of severe orlistat-associated liver injury. Elderly patients with alcohol use and prolonged orlistat exposure were overrepresented among severe cases, suggesting that these factors may warrant particular attention. However, given the small sample size and potential for confounding, these observations are hypothesis-generating. Larger, controlled studies are needed to clarify whether orlistat plays a causal role in severe liver injury and to identify patient subgroups that might benefit from closer monitoring.

## Data Availability

The original contributions presented in the study are included in the article/supplementary material, further inquiries can be directed to the corresponding authors.
